# Transforming Healthcare: Artificial Intelligence (AI) Applications in Medical Imaging and Drug Response Prediction

**DOI:** 10.14293/genint.15.1.002

**Published:** 2025-01-22

**Authors:** Karthik Prathaban, M. Prakash Hande

**Affiliations:** Department of Physiology, Yong Loo Lin School of Medicine, National University of Singapore, Singapore, Singapore

**Keywords:** Artificial intelligence, AI in biomedicine, medical imaging, drug response

## Abstract

Artificial intelligence (AI) offers a broad range of enhancements in medicine. Machine learning and deep learning techniques have shown significant potential in improving diagnosis and treatment outcomes, from assisting clinicians in diagnosing medical images to ascertaining effective drugs for a specific disease. Despite the prospective benefits, adopting AI in clinical settings requires careful consideration, particularly concerning data generalisation and model explainability. This commentary aims to discuss two potential use cases for AI in the field of medicine and the overarching challenges involved in their implementation.

## Computer Vision Applications in Modern Medicine

In recent years, artificial intelligence (AI) models have been rapidly adopted for medical imaging applications. Deep learning models have greatly improved the speed and accuracy of disease diagnosis, from microscopic digital pathology exams to larger-scale radiology evaluations. Early medical image analysis relied on convolutional neural networks (CNNs) as the leading models. Architectures such as ResNet, MobileNet, and Visual Geometry Group showed strong performance in tasks like cancer detection and histopathology subtyping.^[1–6]^ CNN models for image segmentation are capable of accurately delineating structures. For example, the U-net architecture has been shown to accurately demarcate the hippocampus in magnetic resonance imaging scans to predict dementia or outline the lungs in X-rays to diagnose lung diseases.^[7,8]^ In deployment, Viz.AI, a computer vision AI-powered software for stroke detection, has demonstrated significant improvements in both the identification and treatment of large vessel occlusions (LVOs) across multiple comprehensive stroke centres.^[9,10]^ One study showed that the implementation of Viz.AI led to a 95% increase in the transfer rate of LVO patients for mechanical thrombectomy for ischaemic stroke treatment.^[9]^ Another study found that using Viz.AI for patients with basilar artery occlusions resulted in a median improvement of 13 points (IQR 9–24) in National Institutes of Health Stroke Scale (NIHSS) scores from admission to discharge.^[10]^ In comparison, patients without Viz.AI-assisted evaluations showed a median improvement of only 6.5 points (IQR 0–13).^[10]^ In more recent solutions, Vision Transformer (ViT) models are increasingly being explored as alternatives to CNNs for the automated analysis of medical images.^[11]^ ViTs use an attention mechanism initially developed for natural language processing (NLP) to capture relationships across image sections, providing richer contextual information than CNNs.^[11,12]^ This context awareness has shown benefits in analysing microscopic and radiographic images.^[13,14]^ Modern AI solutions in medical image analysis have also leveraged the attention mechanism alongside correlations between encoded representations of images and text to allow for medical image captioning.^[15–17]^ The textual diagnosis output by these models allows for easier interpretation of the model’s findings to the benefit of clinicians.

## Machine Learning for Drug Response Prediction

The treatment of complex diseases, particularly cancers, remains a complex challenge due to tumour heterogeneity, which results in different treatment responses for patients with the same cancer type.^[18,19]^ Predicting the treatment response of different drugs for a specific molecular profile enables the personalization of treatments to improve patient outcomes. Automating this task through machine learning introduces significant advantages in clinical settings but can be disrupted by the high dimensionality of data such as genetic profiles. Classical machine learning approaches, such as random forests, have achieved favourable performance in predicting therapy outcomes when combined with feature selection techniques.^[20]^ To preserve biological information more efficiently, deep learning techniques, such as variational autoencoders, have been used to obtain low-dimensional nonlinear embeddings, effectively capturing patterns and relationships in high-dimensional data.^[21,22]^ As with medical image analysis, recent developments in this context have explored the potential of transformer-based models.^[23,24]^ Using genetic, mutation, or drug information as sequences, the attention mechanism in these models highlights essential data, enhancing response prediction accuracy across various datasets and cancer types.^[23,24]^ Beyond cancer treatment, AI has been instrumental in drug repurposing efforts, such as the discovery of baricitinib as a potential COVID-19 treatment. Using deep learning and biomedical knowledge graphs, researchers identified baricitinib’s potential to inhibit viral infection and reduce inflammation.^[25]^ This finding helped accelerate the drug’s trial process, ultimately leading to emergency Food and Drug Administration authorisation.^[25]^ Examples like this illustrate the capabilities of AI algorithms in identifying effective treatments in a fraction of the traditional timeline.

## Challenges with Adopting AI in Medical Contexts

Given the critical nature of medical decision-making, it is imperative that AI models demonstrate robustness. Methods in ensuring model explainability allow clinicians to understand the rationale behind a model’s outputs. In computer vision, methods such as class activation mapping for CNNs or attention maps for transformers highlight image regions crucial to the model’s output, such as classifications.^[11,26,27]^ These techniques allow clinicians to ensure that a model pays attention to biologically relevant features within the image space to confirm its reliability in diagnoses.^[26,28]^ Exploiting datasets effectively is also a challenge in the medical field. AI model performance depends on access to large, diverse datasets that enable accurate predictions of unseen data. This can be a challenge in the medical field when data is sensitive and patient-derived samples are hard to acquire. Transfer and self-supervised learning enable models to learn basic features from large, generic datasets, improving their performance when fine-tuned on medical data.^[29,30]^ Multi-task learning is another approach to improve performance across multiple tasks using their shared features. In drug response prediction tasks, auxiliary models may be trained alongside response predictors to supplement their ability to gather meaningful features from drug, cell-line or patient data for enhanced performance.^[23,31]^ Despite these technical innovations, some limitations hinder the deployment of AI models in medical facilities. A significant concern is AI algorithms’ potential to exaggerate biases prevalent in their training data. A prominent example of this is the Optum algorithm, a risk prediction algorithm designed to identify high-risk patients for efficient enrolment in healthcare management programmes.^[32]^ The algorithm relied on healthcare costs as an indicator of healthcare needs, which resulted in lower risk scores for Black patients.^[32]^ To prevent scenarios like this from recurring, a careful examination of training data and model outputs must be carried out to identify and eliminate potential biases. It is the ethical responsibility of developers and healthcare professionals adopting AI models in their workflow to ensure that the models are transparent and fair.

Technological advancements in medicine continue to generate an ever-growing amount of data, presenting new and exciting opportunities for AI development. Although this article has focused on two specific use cases, there are numerous challenges and medical data types to which AI techniques can be applied. For instance, NLP can significantly enhance clinical decision-making by extracting and consolidating crucial patient information from electronic health records for diagnostic reference.^[33,34]^ Time series algorithms can be applied to electrocardiograms to identify cardiovascular anomalies.^[35]^ Beyond aiding diagnosis, in-depth data analysis with machine or deep learning algorithms also presents opportunities to discover patterns in biology or diseases that may yield better therapeutic solutions. By improving diagnosis and therapy, AI models may be integral in ensuring better outcomes for patients in the near future.
